# A Suggested New Flag for Hospitals

**Published:** 1914-12-12

**Authors:** F. J. Frankau

**Affiliations:** St. George's Hospital, S.W.


*To the Editor of* The Hospital.

### A Suggested New Flag for Hospitals.

*To the Editor of* The Hospital.

Sir,—The "new" flag suggested for hospitals by Mr. J. B. Cornish in your last issue is nothing more or less than the national flag of Switzerland.

As a matter of fact, the (Geneva Convention) Red Cross flag was arrived at by reversing the colours (a white Greek cross on a red ground) of the Swiss flag.—Yours truly,

F. J. Frankau.

St. George's Hospital, S.W.

